# Polygenic risk-based prediction of heart failure in young patients with atrial fibrillation: an analysis from UK Biobank

**DOI:** 10.1093/europace/euaf104

**Published:** 2025-05-19

**Authors:** Hyo-Jeong Ahn, Tae-Min Rhee, Eue-Keun Choi, Kyung-Yeon Lee, JungMin Choi, Jae-Hyun Kim, Seokmoon Han, Soonil Kwon, So-Ryoung Lee, Seil Oh, Gregory Y H Lip

**Affiliations:** Department of Internal Medicine, Seoul National University Hospital, Republic of Korea; Department of Internal Medicine, Seoul National University Hospital Healthcare System Gangnam Center, Republic of Korea; Department of Internal Medicine, Seoul National University Hospital, Republic of Korea; Department of Internal Medicine, Seoul National University College of Medicine, Republic of Korea; Department of Internal Medicine, Seoul National University Hospital, Republic of Korea; Department of Internal Medicine, Seoul National University Hospital, Republic of Korea; Department of Internal Medicine, Seoul National University Hospital, Republic of Korea; Department of Internal Medicine, Seoul National University Hospital, Republic of Korea; Department of Internal Medicine, Seoul National University Boramae Medical Center, Republic of Korea; Department of Internal Medicine, Seoul National University Hospital, Republic of Korea; Department of Internal Medicine, Seoul National University College of Medicine, Republic of Korea; Department of Internal Medicine, Seoul National University Hospital, Republic of Korea; Department of Internal Medicine, Seoul National University College of Medicine, Republic of Korea; Department of Internal Medicine, Seoul National University College of Medicine, Republic of Korea; Liverpool Centre for Cardiovascular Science at University of Liverpool, Liverpool John Moores University and Liverpool Chest and Heart Hospital, Liverpool, UK; Department of Clinical Medicine, Aalborg University, Aalborg, Denmark

**Keywords:** Polygenic risk score, Atrial fibrillation, Heart failure, Age, Genetic risk

## Abstract

**Aims:**

Heart failure (HF) is the most concerning morbidity in atrial fibrillation (AF) through mutual influence on a poor prognosis. A polygenic risk score (PRS) has recently been proposed to improve the risk prediction for cardiovascular disease. The additive predictive role of PRS for incident HF in patients with AF who inherently carry a high risk of HF is unknown.

**Methods and results:**

From the UK Biobank, we identified 21 167 White Caucasian participants with newly diagnosed AF without a prior history of HF. The PRS for HF was constructed using genetic instruments from previous genome-wide association studies. The primary outcome was the occurrence of incident HF. The prediction of incident HF was evaluated using the tertile categorization of PRS for HF (low vs. moderate-high PRS) across the entire AF cohort, as well as within age subgroups (young AF, age <60 years; old AF, age ≥60 years, respectively). The mean age was 69.0 ± 6.9 years in the total population (55.2 ± 3.9 years in age <60 years; 70.7 ± 5.0 years in age ≥60 years group). During a median follow-up of 3.8 (1.4–7.2) years, the incidence rate (1000-patient year) of HF was 29.9. In the total population, AF patients with moderate-high PRS for HF were associated with a higher risk of HF than those with low PRS for HF [adjusted hazard ratio (HR) 1.18 (95% confidence interval (CI), 1.05–1.32), *P* = 0.005]. The higher risk of HF in the moderate-high PRS group was particularly accentuated in young AF patients: adjusted HR, 2.14 (95% CI 1.29–3.57) in young AF, and 1.13 (95% CI 1.01–1.27) in old AF, *P*-for-interaction = 0.015. In young AF, the onset of incident HF was earlier in those with the moderate-high PRS group [median time from AF diagnosis to incident HF, 4.2 (0.8–7.1) years in low PRS vs. 1.5 (0.3–4.7) years in the moderate-high PRS group, *P* = 0.001]. The prediction of HF was significantly improved by adding PRS to the clinical risk factors for HF, especially in young AF patients, with a net reclassification improvement of 29.7% (*P* = 0.003).

**Conclusion:**

PRS for HF can significantly improve the prediction of incident HF in patients with AF, especially in the young population, providing clinical utility of an individualized approach to integrated management of AF.

## Graphical Abstract

**Graphical Abstract:**
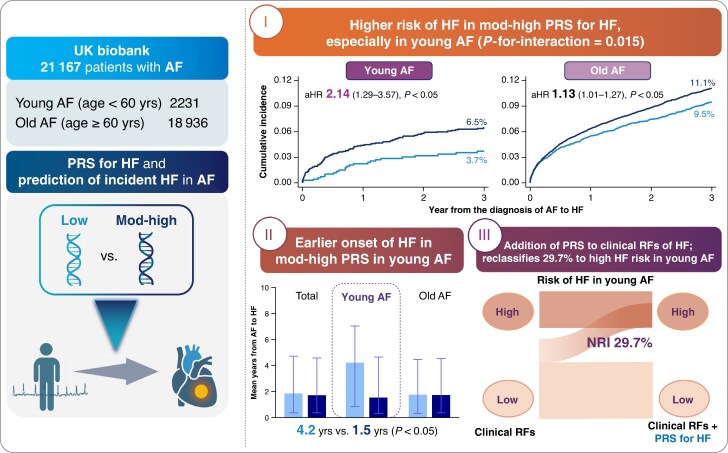

## Introduction

Atrial fibrillation (AF), the most common sustained cardiac arrhythmia, is associated with a five-fold increased risk of heart failure (HF).^1–3^ AF and HF are closely related by sharing common risk factors^4^; ∼40% of patients with either AF or HF are likely to develop the other condition.^5^ The prevalence of both AF and HF is increasing,^6^ and unfortunately, subsequent development of HF in patients with AF is associated with a much worse prognosis.^5^ Hence, it is imperative to identify and predict the elevated risk of HF in patients with AF, intervene early, and halt the mutual progression of each disease.

Over the past decade, genome-wide association studies (GWAS) have increasingly confirmed the polygenic basis—i.e. the contribution of many single nucleotide variants (SNVs)—of various cardiometabolic diseases.^7^ A polygenic risk score (PRS) is the summation of SNVs across the genome into a single score. It is shown to be independently associated with cardiometabolic diseases such as AF, coronary artery disease (CAD), or diabetes mellitus (DM).^7–10^ Recent studies suggest that PRS improves prediction for the development of cardiometabolic diseases when added to existing clinical risk factors.^11^ In terms of HF, the additive role of PRS in predicting incident HF was only modest in the general population due to phenotype heterogeneity and changing disease definitions.^11^ Meanwhile, the additive predictive role of PRS for incident HF in high risk groups, such as in patients with AF, is unknown.

Several prediction models of incident HF or HF hospitalization in patients with AF have been suggested,^12–14^ but none have integrated the genetic pre-disposition, as reflected by a PRS, to HF thus far. Moreover, little is known about HF prediction in AF when stratified by age. Since young AF patients, those diagnosed with AF before the age of 60,^15–18^ are likely to have distinct pathophysiology compared to older AF patients (i.e. diagnosed at 60 years or older) as well as being exposed to a longer lifetime risk of morbidity, it would be important to evaluate the risk and improve prediction of HF in this population.

From a prospective cohort of European ancestry patients with AF, we aimed to evaluate the following: (i) the ability of PRS to categorize the risk of HF in AF; (ii) the variations in risk categorization according to age group; and (iii) the extent of enhancement in predictive accuracy for HF by incorporating PRS alongside established clinical risk factors.

## Method

### Study participants

**Figure 1:**
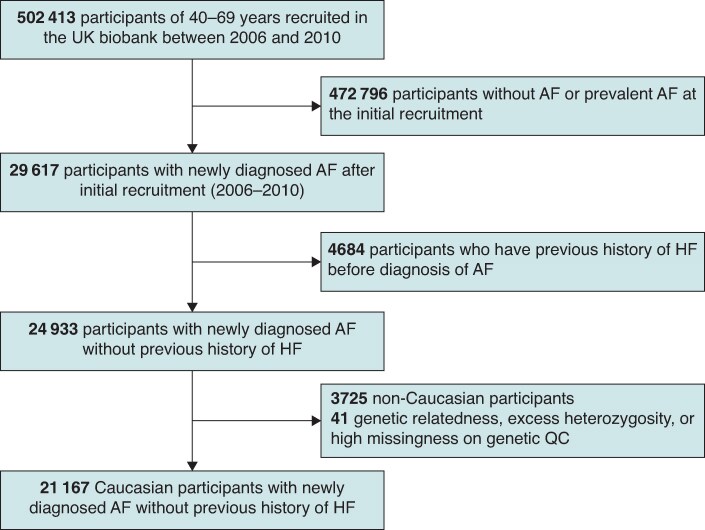
Inclusion of study population. Abbreviations: AF, atrial fibrillation; HF, heart failure; QC, quality control.

The UK Biobank is a comprehensive longitudinal cohort study that enrolled over 500 000 individuals aged between 40 and 69 during 2006–10. Individuals were registered with the UK National Health Service and resided within 25 miles of an assessment centre. The present study acquired approval from the UK Biobank’s review committee under application number 76593 and ethical approval by the North West Multi-Centre Research Ethics Committee. The entire protocols are detailed in other publications.^19^ Written informed consent was obtained from all participants. We utilized data from the UK Biobank, including self-reported questionnaires, measurements of body metrics, and disease diagnosis information from hospital and death registries. From 502 413 initially eligible participants, we excluded individuals with prevalent AF at the time of initial recruitment, as well as those who did not receive an AF diagnosis during the cohort follow-up. Consequently, our study included only participants who were newly diagnosed with AF after their initial recruitment. We then excluded individuals with a history of HF before AF diagnosis, those of non-European descent, and individuals with genetic relatedness, excessive heterozygosity, or high missingness in genetic quality control. The final cohort for analysis comprised 21 167 patients with AF (*Figure 1*). The ethical approval for this study was provided by the National Health Service National Research Ethics Service on 17 June 2011 (Ref 11/NW/0382), with an extension granted on 10 May 2016 (Ref 16/NW/0274).

### Genotyping

Genotyping for each participant was performed as described elsewhere,^20^ employing either the UK Biobank Lung Exome Variant Evaluation Axiom array (Affymetrix; *n* = 49 950; including 807 411 single nucleotide polymorphisms [SNPs]) or the UK Biobank Axiom array by Affymetrix (Affymetrix; *n* = 438 427; including 825 927 SNPs). Imputation of genotype data were conducted using reference panels from the Haplotype Reference Consortium, UK10K, and 1000 Genomes Phase 3 panels.^21–23^ For further analyses, SNPs not in Hardy–Weinberg equilibrium (*P* < 1.0 × 10^−6^), with minor allele frequency <1%, or with genotyping failure rate >5% were excluded.

### Genome-wide association study summary statistics to calculate polygenic risk scores

We constructed PRS for HF using GWAS summary statistics from the HEart failure Molecular Epidemiology for Therapeutic targetS (HERMES) consortium. Using the MetaSubtract package in R, we adjusted for the influence of GWAS data from the UK Biobank itself, utilizing summary statistics for 8 280 706 variants from 583 167 residual participants.^24^

To avoid inflated results due to a duplicated population in the discovery cohort used for GWAS and the validation cohort for this study, we utilized the MetaSubtract method that derives summary statistics of a meta-GWAS (summary statistics for 8 280 706 variants from 583 167 residual participants), leaving the UK Biobank cohort out.^24,25^

### Calculating polygenic risk scores using the continuous shrinkage method

The PRS was calculated using the continuous shrinkage (PRS-CS) approach, which estimates posterior effect sizes of SNPs from GWAS summary statistics and linkage disequilibrium data from external reference panels.^26^ By applying Bayesian regression with continuous shrinkage priors, we determined the effect size for each SNP. The phi parameter was automatically chosen using the PRS-CS-auto method. The PRS for each participant was the sum of these effects, normalized by the total number of SNPs. The beta coefficient and *P* values of each SNP for each outcome were taken from the corresponding summary statistics data.

### Covariates

AF and HF were defined in the death registry and inpatient hospital registry data incorporated in the UK Biobank, using the corresponding International Classification of Diseases (ICD)-10 codes (for AF, I48; for HF, I50, I11.0, I13.0, and I13.2). Detailed definitions of covariates and outcomes are described in Supplementary material online, *Table S1*. The covariates included age, sex, body mass index (BMI), enrolment centre, household income before tax, Townsend deprivation index (TDI), current smoking, daily drinking, previous history of DM, hypertension, myocardial infarction (MI), stroke, or any cardiomyopathies, taking medications for hypertension and dyslipidaemia, genotype array, and genetic principal component (PC) 1 to 10.

### Study outcome

The primary outcome was the occurrence of incident HF during the follow-up period after diagnosis of AF. PRS for HF was stratified according to tertile groups [low PRS with 1st tertile vs. moderate(mod)-high PRS with 2nd and 3rd tertile]. The risk of primary outcome was compared between AF patients with low PRS and mod-high PRS for HF. The analyses were stratified by age group determined by the cut-off of 60 years, as specified in previous epidemiologic and genetic landmark research of early onset AF (young AF, <60 years; old AF, ≥60 years, respectively).^15–18^ Participants were censored on the earliest date, either the date of the end of follow-up, the outcome occurrence, or death.

### Statistical analyses

Continuous variables were presented as means with standard deviations (SD), and categorical variables as frequencies and percentages. The χ^2^ and Student’s *t*-tests were used for categorical and continuous variables, respectively. The Kaplan–Meier estimated cumulative incidence of HF was calculated and compared between the tertile groups of PRS for HF using the log-rank test. We used multi-variable Cox proportional hazard models to estimate hazard ratios (HRs) and 95% confidence intervals (CIs) according to low PRS vs. mod-high PRS for HF. The time variable computed in the Kaplan–Meier survival analysis and multi-variable Cox proportional hazard model was defined as the duration from AF diagnosis (index date) to the first occurrence of HF, the end of follow-up date, or death. The covariates used for adjustment included age, sex, enrolment centre, BMI, income level, hypertension, DM, MI, stroke, any diagnosis of cardiomyopathies, genotype array, and the 1st to 10th genetic PCs, which presented differences between low PRS and mod-high PRS for HF group. The enhancement of the predictive ability by adding genetic risk was assessed by Harrell’s C-statistics and category-free net reclassification improvement (NRI), comparing a prediction model based on clinical risk factors selected from stepwise Cox regression analysis and a model that also included PRS for HF. The time from AF diagnosis to HF onset was also analysed across groups using a *t*-test.

Subgroup analyses for the risk stratification of HF by PRS (low vs. mod-high PRS) in AF were performed according to sex, hypertension, DM, and history of MI. To confirm the robustness of our finding, we conducted a sensitivity analysis in two ways: (i) by excluding AF patients with any diagnosis of cardiomyopathies throughout the study period, and (ii) by excluding the incident HF that occurred within the first 30 days and 90 days of follow-up. A *P*-value <0.05 was considered statistically significant. All statistical analyses were performed using R software (v4.2.3) and Stata software (v18.0).

## Results

### Baseline characteristics

The baseline characteristics of the study population stratified by age are described in *Table 1*. Among the total 21 167 patients with newly diagnosed AF without previous history of AF, there were 2231 (10.5%) patients categorized as ‘young AF’ (age at AF diagnosed <60 years) and 18 936 (89.5%) patients categorized as ‘old AF’ (age ≥60 years).

**Table 1:** Baseline characteristics of patients with atrial fibrillation included in the study

	Total	Age < 60 years	Age ≥ 60 years	*P*-value
	*N* = 21 167	*N* = 2231 (10.5%)	*N* = 18 936 (89.5%)	
Age (years)	69.0 ± 6.9	55.2 ± 3.9	70.7 ± 5.0	<0.001
Male	12 907 (61.0%)	1478 (66.2%)	11 429 (60.4%)	<0.001
BMI (kg/m^2^)	28.8 ± 5.3	29.3 ± 6.1	28.7 ± 5.2	<0.001
Household income before tax (pound)				<0.001
<18 000	5577 (26.3%)	343 (15.4%)	5234 (27.6%)	
18 000–30 999	5107 (24.1%)	376 (16.9%)	4731 (25.0%)	
31 000–51 999	3731 (17.6%)	585 (26.2%)	3146 (16.6%)	
52 000–100 000	2279 (10.8%)	544 (24.4%)	1735 (9.2%)	
>100 000	580 (2.7%)	150 (6.7%)	430 (2.3%)	
Comorbidities				
Hypertension	13 299 (62.8%)	965 (43.3%)	12 334 (65.1%)	<0.001
Myocardial infarction	2323 (11.0%)	163 (7.3%)	2160 (11.4%)	<0.001
Diabetes mellitus	3104 (14.7%)	261 (11.7%)	2843 (15.0%)	<0.001
Stroke	1911 (9.0%)	113 (5.1%)	1798 (9.5%)	<0.001
Cardiomyopathy	211 (1.0%)	37 (1.7%)	174 (0.9%)	0.001
Medications				
Hypertension medications	8348 (39.4%)	475 (21.3%)	7873 (41.6%)	<0.001
Dyslipidaemia medications	6657 (31.4%)	356 (16.0%)	6301 (33.3%)	<0.001
Lifestyle behaviours				
Current smoking	2207 (10.4%)	339 (15.2%)	1868 (9.9%)	<0.001
Daily drinking	5091 (24.6%)	447 (20.4%)	4644 (25.1%)	<0.001
Townsend deprivation index	−1.4 ± 3.0	−1.1 ± 3.2	−1.4 ± 3.0	<0.001
CHA₂DS₂-VASc	2.4 ± 1.4	1.1 ± 1.0	2.6 ± 1.3	<0.001

The mean age of the total AF population was 69.0 ± 6.9 years: young AF, 55.2 ± 3.9 years, and old AF, 70.7 ± 5.0 years. Overall, the proportion of males was 61.0%, with a higher proportion in the young AF category than old AF (66.2% vs. 60.4%, *P* < 0.001). Various comorbidities—hypertension, MI, DM, and stroke—were more common in old AF (all *P* < 0.001). On the other hand, the prevalence of cardiomyopathy was higher in young AF than in old AF (1.7% vs. 0.9%, *P* < 0.001). The proportion of current smoking was higher in young AF, whereas those with daily drinking were higher in old AF.

Baseline characteristics of the total population, young AF, and old AF stratified by the PRS for HF (low PRS vs. mod-high PRS) are presented in Supplementary material online, *Table S2*. In total, 7056 (33.3%) patients with AF had low PRS for HF and 14 111 (66.7%) patients with AF had mod-high PRS for HF. The proportion of mod-high PRS for HF was significantly higher in the young AF category than in old AF (68.6% vs. 66.4%, *P* = 0.038). In both age categories, those with mod-high PRS for HF had significantly higher BMI and a greater prevalence of DM than those with a low PRS for HF (*P* < 0.05).

### Polygenic risk-based prediction of heart failure in atrial fibrillation

**Figure 2:**
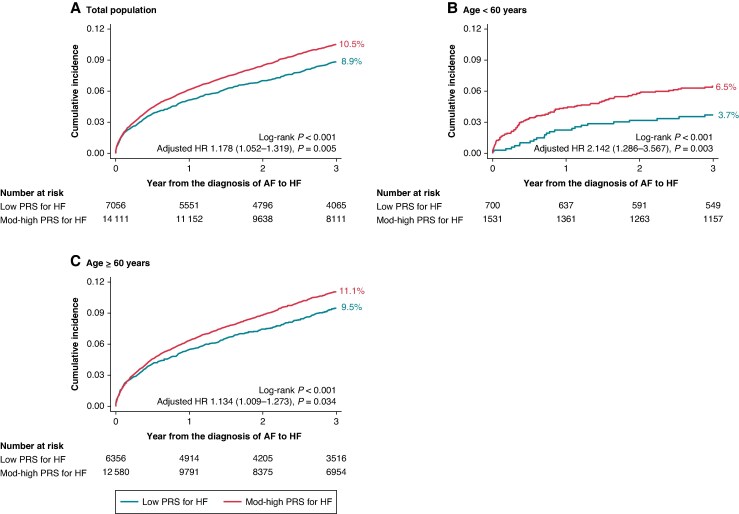
The cumulative risks of HF according to the PRS in patients with atrial fibrillation. *A*) Total population, *B*) patients with age < 60 years, and *C*) patients with age ≥ 60 years. Abbreviations: HR, hazard ratio; AF, atrial fibrillation; HF, heart failure; PRS, polygenic risk score; Mod, moderate.

The risk of HF in patients with AF according to the PRS for HF is presented in *Table 2* and *Figure 2*. During a median follow-up of 3.8 (inter-quartile range, 1.4–7.2) years, the incidence rate for HF per 1000 patient-year (PY) was 29.85 (low PRS, 27.02; mod-high PRS, 31.25 per 1000-PY) for total AF population, 14.32 (low PRS, 12.14; mod-high PRS, 15.35 per 1000-PY) for the young AF category, and 32.63 (low PRS, 29.61; mod-high PRS, 34.13 per 1000-PY) for old AF.

**Table 2:** The risk of heart failure according to the polygenic risk score in patients with atrial fibrillation stratified by age

Group	Event/*N*^a^	Cumulative incidence^b^	Adjusted HR (95% CI)^b^		
			Model 1	Model 2	Model 3
Total					
Low PRS for HF	855/7056	8.9%	1 (Reference)	1 (Reference)	1 (Reference)
Mod-High PRS for HF	2003/14 111	10.5%	1.201 (1.084–1.329)	1.215 (1.097–1.345)	1.178 (1.052–1.319)
			*P* < 0.001	*P* < 0.001	*P* = 0.005
Age < 60					
Low PRS for HF	57/700	3.7%	1 (Reference)	1 (Reference)	1 (Reference)
Mod-High PRS for HF	151/531	6.5%	1.881 (1.199–2.951)	1.850 (1.179–2.902)	2.142 (1.286–3.567)
			*P* = 0.006	*P* = 0.007	*P* = 0.003
Age ≥ 60					
Low PRS for HF	798/6356	9.5%	1 (Reference)	1 (Reference)	1 (Reference)
Mod-High PRS for HF	1852/12 580	11.1%	1.174 (1.057–1.303)	1.184 (1.066–1.315)	1.134 (1.009–1.273)
			*P* = 0.003	*P* = 0.002	*P* = 0.034
			*P*-for-interaction = 0.063	*P*-for-interaction = 0.071	*P*-for-interaction = 0.015

Model 1 was adjusted by chip and genetic principal component 1–10.

Model 2 was adjusted by age, sex, enrolment centre, chip, and genetic principal component 1–10.

Model 3 was adjusted by age, sex, enrolment centre, BMI, household income before tax, hypertension, myocardial infarction, diabetes mellitus, stroke, any cardiomyopathy, chip, and genetic principal component 1–10.

AF, atrial fibrillation; CI, confidence interval; HF, heart failure; HR, hazard ratio; PRS, polygenic risk score.

^a^Observed events during total follow-up period.

^b^3-Year cumulative incidence and HRs.

The 3-year cumulative incidence of HF was higher in the mod-high PRS group than in the low PRS group in both age categories: i.e. low vs. mod-high PRS, 8.9% vs. 10.5% in total AF; 3.7% vs. 6.5% in young AF, and 9.5% vs. 11.1% in old AF, respectively (all log-rank *P* < 0.001, *Figure 2*). Overall, the risk of HF was ∼1.18-fold higher in patients with mod-high PRS than those with low PRS for HF after multi-variable adjustment (model 3): adjusted HR (95% CI) 1.178 (1.052–1.319), *P* = 0.005. The higher risk of HF in AF patients with mod-high PRS was greater among young AF than old AF; adjusted HR (95% CI) of HF in mod-high PRS group vs. low PRS group was 2.142 (1.286–3.567), *P* = 0.003 in young AF, whilst in old AF, adjusted HR (95% CI) was 1.134 (1.009–1.273), *P* = 0.034 (*P*-for-interaction = 0.015) (*Table 2*). When analysed as a continuous variable, a one-SD increase in HF PRS was significantly associated with a 29.0% higher risk of HF in young AF, whereas an 8.2% increase in old AF (see Supplementary material online, *Table S3*).

### Moderate to high polygenic risk score and early onset of heart failure

**Figure 3:**
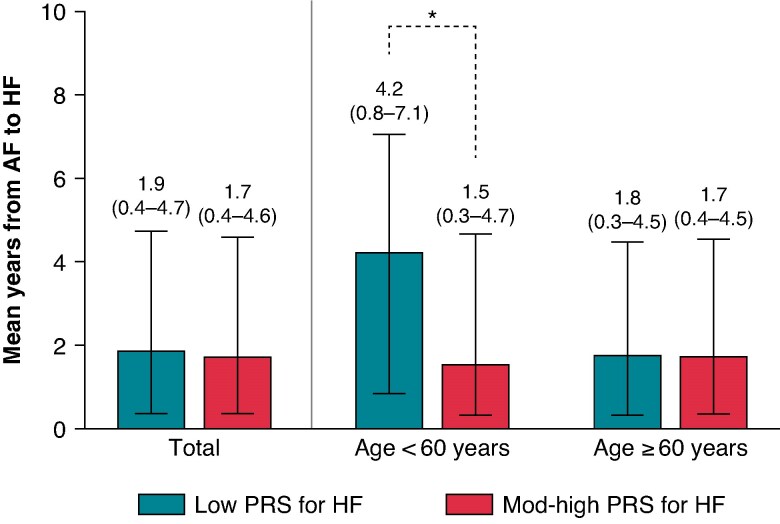
PRS and the onset of HF in patients with atrial fibrillation. Abbreviations: AF, atrial fibrillation; HF, heart failure; PRS, polygenic risk score; Mod, moderate. * *P* < 0.05.

A shorter duration from the diagnosis of AF to the onset of HF (incident HF) in those with mod-high PRS for HF than those with low PRS for HF was evident in young AF. Among young AF, the median years from AF diagnosis to incident HF was 4.2 (0.8–7.1) years in those with low PRS for HF and 1.5 (0.3–4.7) years with mod-high PRS for HF (*P* = 0.001). There were no significant differences between two groups in the old AF category (*Figure 3*).

### Improvement in prediction of heart failure with the integration of polygenic risk scores

The additive role of PRS in predicting the risk of HF in patients with AF was evaluated by Harrell’s C-statistics and NRI and described in *Table 3*.

**Table 3:** Harrell’s C-statistics and net reclassification improvement of polygenic risk score in predicting heart failure in patients with atrial fibrillation

	Harrell’s C-statistics (95% CI)	*P*-value	NRI (%)	*P*-value
Total				
Model A	0.638 (0.624–0.653), *P* < 0.001	–	Reference	–
Model A + PRS for HF (low vs. mod-high)	0.640 (0.626–0.655), *P* < 0.001	0.091	8.86 (3.51–14.21)	0.001
Age <60				
Model B	0.690 (0.638–0.741), *P* < 0.001	–	Reference	–
Model B + PRS for HF (low vs. mod-high)	0.714 (0.669–0.758), *P* < 0.001	0.020	29.74 (10.26–49.22)	0.003
Age ≥60				
Model C	0.629 (0.615–0.642), *P* < 0.001	–	Reference	–
Model C + PRS for HF (low vs. mod-high)	0.631 (0.617–0.644), *P* < 0.001	0.039	7.8 (2.76–12.84)	0.002

Clinical features of Model A, B, and C to predict heart failure in patients with atrial fibrillation were selected from stepwise Cox regression analysis in each age group (total vs. age <60 years vs. age ≥60 years) among the following covariate: age, sex, BMI, household income before tax, hypertension, myocardial infarction, diabetes mellitus, stroke, any cardiomyopathy, current smoking, daily drinking, hypertension medication, dyslipidaemia medication, and Townsend Deprivation Index.

Model A: age, male, BMI, household income before tax, hypertension medication, myocardial infarction, diabetes mellitus, any cardiomyopathy, Townsend Deprivation Index, and current smoking.

Model B: male, BMI, household income before tax, myocardial infarction, hypertension medication, and dyslipidaemia medication.

Model C: age, male, BMI, hypertension, myocardial infarction, diabetes mellitus, any cardiomyopathy, current smoking, and Townsend Deprivation Index.

CI, confidence interval; HF, heart failure; mod, moderate; NRI, net reclassification improvement; PRS, polygenic risk score.

In the total population, clinical risk factors to be associated with incident HF selected from stepwise cox-regression analysis were age, sex, BMI, household income, hypertension medications, MI, DM, cardiomyopathy, current smoking, and TDI. A collection of these clinical risk factors has a C-statistic 0.638 (0.624–0.653), and the addition of PRS resulted in a minimal increment of 0.640 (0.626–0.655), with the comparison *P*-value 0.091. The inclusion of PRS significantly increased the NRI compared to the summation of clinical risk factors: NRI, 8.86 (3.51–14.21) %, *P* = 0.001.

The increased predictive accuracy of HF was greater in young AF. Stepwise Cox-regression analysis identified sex, BMI, household income, hypertension medication, dyslipidaemia medication, and MI as clinical risk factors associated with incident HF in young AF. Adding PRS to the summation of clinical risk factors significantly increased C-statistics from 0.690 (0.638–0.741) to 0.714 (0.669–0.758) with a *P*-value of 0.020. The NRI for HF with PRS was 29.74 (10.26–49.22) %, *P* = 0.003, indicating a greater improvement in HF prediction in young AF.

In old AF, the additive predictive role of PRS in HF was modest but significant. Stepwise Cox-regression analysis demonstrated age, sex, BMI, hypertension, MI, DM, cardiomyopathy, current smoking, and TDI as related clinical risk factors to an incident HF. The PRS improved the prediction of HF in AF, resulting in an increment of C-statistic from 0.629 (0.615–0.642) to 0.631 (0.617–0.644), *P* = 0.039 and NRI 7.8 (2.76–12.84) %, *P* = 0.002.

The discrete categorized reclassification analysis is detailed in Supplementary material online, *Table S4*. After stratifying the study population into 7 risk categories (<2.5%, 2.5 to <5%, 5% to <7.5%, 7.5% to <10%, 10% to <12.5%, 12.5% to <15%, and ≥15% of predicted 3-year HF risk), the addition of PRS-based genetic risk group on the clinical risk factor model in each age group substantially improved the reclassification performance of the model with estimated category-based NRI of 20.4% (95% CI 6.7–34.1%, *P* = 0.004) only in the young AF group.

### Subgroup and sensitivity analyses

In the total population, subgroup analyses consistently showed a higher risk of HF in AF patients with mod-high PRS than low PRS irrespective of sex, hypertension, and DM (*P*-for-interaction > 0.05). Of note, the risk of HF was not stratified by PRS in AF patients with MI (*P*-for-interaction = 0.042) (see Supplementary material online, *Table S5*).

Subgroup analyses were also performed in young AF. Compared to the total AF population, young AF patients with mod-high PRS for HF presented a higher risk of HF than low PRS for HF across all subgroups (all *P*-for-interaction > 0.05). The augmented increased risk of mod-high PRS for HF in young AF than that of old AF was consistent in males and those without hypertension, DM, or MI (*P*-for-interaction, in comparison with adjusted HR of mod-high PRS in old AF <0.05) (see Supplementary material online, *Table S5*).

Sensitivity analyses were performed by excluding AF patients with prevalent (*n* = 211) and incident cardiomyopathy (*n* = 304). The higher risk of HF in AF patients with mod-high PRS and amplified increased risk in young AF was maintained after excluding AF patients with cardiomyopathy (see Supplementary material online, *Table S6*). The improvement in the prediction of HF by adding PRS to the clinical risk factors was also prominent in young AF, with an increment of C-statistic from 0.704 to 0.734, *P* = 0.012 and NRI 33.44, *P* = 0.002 (see Supplementary material online, *Table S7*). Additional sensitivity analysis was performed by excluding the incident HF that occurred within the first 30 days (see Supplementary material online, *Table S8*) and 90 days (see Supplementary material online, *Table S9*) of follow-up. The increased risk of HF in AF patients with a mod-high PRS remained significant after applying a 30-day and 90-day blanking period to exclude early incident HF cases during follow-up. The patients with AF of mod-high PRS presented 1.878-fold (30-day blanking period) or 1.763-fold (90-day blanking period) higher risk of HF in young AF, whereas 1.176-fold (30-day blanking period) or 1.179-fold (90-day blanking period) higher risk of HF in old AF (*P*-for-interaction 0.097 for 30-day and 0.167 for 90-day blanking period, respectively).

## Discussion

From a European ancestry-prospective white Caucasian cohort of patients with AF, our major findings are summarized as follows: (i) PRS can discriminate the risk of HF in AF with those mod-high PRS associated with a 17.8% higher risk of HF than low PRS group; (ii) increased risk of HF in AF in those with mod-high PRS group was more accentuated in young AF than old AF (2.14-fold vs. 1.13-fold); and (iii) the addition of PRS for HF to the clinical factors predicting HF reclassifies a sizable proportion of patients with AF into high HF risk group, particularly in the young AF category (8.9% in the total AF cohort and 29.7% in young AF).

To the best of our knowledge, this is the first report that validated the adjunctive role of PRS in predicting HF in high-risk groups, namely, in patients with AF. Our findings suggest that a partial genetic basis of HF in AF is present, especially in the young AF population.

Previous research has identified several demographic factors and clinical to be integrated into the HF risk prediction model; age, hypertension, diabetes, history of heart failure/CAD, smoking, poor renal function, BMI, and serum brain natriuretic peptide are representative indicators that are independently associated with the increased risk of HF in AF.^12–14,27^ In agreement, we acknowledged that similar clinical factors—age, BMI, HTN, DM, history of MI, and smoking—are as relevant for incident HF in a large cohort of patients with AF (comprising over 21 000 individuals, significantly surpassing the maximum of ∼3000 patients in prior reports). In our study, we first identified that PRS was an independent risk marker of HF in AF. By integrating the relative genetic risk for HF, we re-classified a sizable portion of AF patients to a group with a high risk of HF. Given the role of genetic factors in arrhythmia has been increasingly elucidated,^28^ our study takes this understanding a step further by demonstrating that genetic factors can more accurately predict the disease course of arrhythmia (i.e. occurrence of HF in AF). This finding not only advances our understanding of the evolving role of genetics in the onset of arrhythmia but also broadens the scope of research to elucidate its impact on the overall progression of arrhythmic disease.

While we show a partial genetic basis of HF in AF, the role of PRS in predicting HF in overall AF patients with MI became non-significant, implying that the application of PRS to predict HF can be used in certain subgroups, but perhaps not those who are in the extremely high clinical risk category. Meanwhile, our analysis confirmed the accentuated predictive role of PRS on HF in young AF. The young AF category is of particular interest given that their numbers are increasing related to changes in lifestyle or obesity, comorbidities and longer lifetime exposure to cardiovascular risks.^29,30^ With the progressive nature of AF,^31,32^ early risk assessment and intervention would result in a favourable prognosis.^33^ In our study, the young AF category with a relative mod-high genetic risk of HF had a 2.1-fold higher risk of HF and an earlier onset of HF compared to those with low genetic risk. Moreover, we showed that nearly one-third of young AF are re-classified to high HF risk, as evidenced by the NRI. We additionally pinpointed specific subgroups within young AF—male or those without hypertension, diabetes, or MI—showing a heightened risk of HF based on their relatively elevated genetic pre-disposition to HF compared to old AF. Thus, the supplementary role of PRS in predicting HF appears to be particularly advantageous for relatively young and medically stable AF. Not only did we verify the genetic risk of incident HF in AF, but we studied the subgroups where implementation of PRS in clinical practice could provide risk refinement to detect HF early and facilitate a more personalized management strategy as needed.

There is a growing body of evidence supporting the utilization of PRS to predict the development and complications of cardiometabolic diseases.^7,11,34,35^ Meanwhile, the performance of PRS for HF in the general population was comparatively lower than for other cardiometabolic diseases, ranging from 1.01 to 1.08 in the UK Biobank data.^36^ This is partly explained by the heterogeneity in the phenotype or aetiology and changing definitions of HF.^11^ On the other hand, there was improved PRS prediction for meticulously defined HF (i.e. dilated cardiomyopathy ascertained by cardiac magnetic resonance imaging) and hypertrophic cardiomyopathy that is more phenotypically distinct.^37,38^

To enhance the predictive role and utility of PRS for HF, an effort should be made to identify a population of interest that can derive the most benefit from it and precisely delineate the phenotype of HF in the context of absolute and relevant clinical risk.^34^ In agreement, our findings from over 21 000 patients with AF who inherently carry a high risk of HF, shows that AF patients are a meaningful subset that PRS further refine risk stratification. Although the C-statistics were only modestly improved (2.4–3.0%), the NRI is notable in the young AF category, thereby suggesting a subgroup that PRS for HF can have clinical utility.^39–41^ In addition, future studies integrating multiple PRS—including recently established PRS for dilated cardiomyopathy,^42^ and PRS for cardiac structural and functional traits—may provide a more comprehensive genetic risk stratification framework for HF prediction in AF patients. Such an approach may enable a more inclusive genetic risk assessment, as distinct PRS capture different pathophysiological mechanisms underlying HF. This aligns with the complex and heterogeneous nature of HF in AF patients, underscoring the potential of multi-PRS models to enhance precision medicine strategies in AF. Based on our research, the PRS for HF could be proposed as a powerful risk marker for integration into machine learning algorithms, a novel approach in predictive systems, especially in the young AF population.^43^

The Framingham Heart Study reported that incident HF in patients with AF is associated with a nearly three-fold increased risk of all-cause mortality.^5^ HF is the most common complication and leading cause of mortality in AF, becoming the most concerning outcome in AF.^44^ Therefore, an enhancement in the prediction of high-risk and early onset HF and facilitating management strategies, including healthier lifestyle and rigorous risk factor control will help achieve improved clinical prognosis and alleviate the healthcare burden associated with HF.^45^ Genetic characterization can be proposed as an approach to stratify patients with AF into different risk trajectories for HF. The genetic risk-based individualized management of AF—i.e. more intensive monitoring for detecting the early sign of HF or more effort to achieve guideline adherent AF care^46–48^—will enable holistic AF treatment, thereby improving overall clinical prognosis.

### Limitations

Our study has several limitations. First, the age cut-off to divide the PRS for HF into two groups was at the authors’ discretion. Therefore, different age thresholds would result in different effect estimates. Second, rare monogenic variants known to be strongly associated with cardiomyopathy or HF are not included in calculating the PRS for HF; therefore, the effect could not be integrated. To evaluate the impact of the summation of common variants solely, we performed sensitivity analysis by excluding prevalent and incident cardiomyopathy patients and confirmed that the additive predictive role of PRS in HF is consistent. Third, systolic function data of incident HF was unavailable and subgroup analysis in accordance to the type of HF could not be performed, requiring further study specifying HF type and aetiology. Fourth, our result might include reverse causality bias which may affect the true temporality of AF and HF since the operational definitions of AF and HF are based on billing codes. To address this, we conducted sensitivity analyses applying a 30-day and 90-day blanking period to exclude early incident HF. The results reinforced the trend of elevated HF risk in AF patients with mod-high PRS, particularly in young AF, but should be further validated in a larger cohort. Fifth, the role of PRS in other ethnic groups needs to be established and externally validated. Sixth, the operational definition of covariates and outcomes might not fully capture participants’ actual disease status. Lastly, the mechanism(s) that can explain the differences in the predictive value of PRS for HF between young and old AF is hypothetical (i.e. different genetic basis) and might not be fully answered. Further studies are required to define the eligible patients with AF for intensified preventive care for HF and to evaluate the effect on clinical outcomes associated with the prospective implementation of PRS.

## Conclusion

The genetic risk for HF can differentiate the risk of incident HF in patients with AF, which is a population at high risk for HF. This would adjunctively improve the prediction of HF than the clinical risk factors. The risk assessment and predictive improvement in HF through PRS was greater in the young AF category. It underscores the genetic basis of HF in young AF patients and highlights the potential clinical utility of personalized prognosis evaluation based on genetic risk and the necessity of tailored management, especially in young AF.

## Supplementary Material

euaf104_Supplementary_Data

## Data Availability

UK Biobank data are publicly accessible to approved researches (https://www.ukbiobank.ac.uk/). The data of this study will be shared on reasonable request to the corresponding author. The authors declare that all illustrations and figures in the manuscript are entirely original and do not require reprint permission. The authors do not have any direct contact information of the study participants because of anonymized data used in accordance with strict confidentiality guidelines. No patient was involved in developing the hypothesis, objectives, or plans for the study’s design. No patient was involved in the interpretation or writing of the results. The authors do not plan to disseminate the results to the individual study participants.
