# Supplementary material for: Polygenic risk-based prediction of heart failure in young patients with atrial fibrillation: an analysis from UK Biobank
Source: Europace. 2025 May 19;27(7):euaf104. doi: 10.1093/europace/euaf104 (PMC12212053; doi:10.1093/europace/euaf104)
Supplement: euaf104_Supplementary_Data [file euaf104_supplementary_data.docx]

**Supplementary materials**

**Supplementary Table 1.** Definitions of covariates and outcomes.

**Supplementary Table 2.** Baseline characteristics of patients with atrial fibrillation included in the study stratified by age and polygenic risk score for heart failure.

**Supplementary Table 3.** The risk of heart failure according to the increment of one-standard deviation of heart failure polygenic risk score in patients with atrial fibrillation stratified by age.

**Supplementary Table 4**. Reclassification of predicted three-year risk of heart failure by adding genetic risk group on clinical risk factor model.

**Supplementary Table 5.** Subgroup analysis of the risk of heart failure according to the polygenic risk score in patients with atrial fibrillation.

**Supplementary Table 6.** The risk of heart failure according to the polygenic risk score in patients with atrial fibrillation, excluding cardiomyopathies stratified by age.

**Supplementary Table 7.** Harrell's C-statistics and net reclassification improvement of polygenic risk score in predicting heart failure in patients with atrial fibrillation, excluding cardiomyopathies.

**Supplementary Table 8.** The risk of heart failure according to the polygenic risk score in patients with atrial fibrillation, excluding the incident heart failure that occurred within the first 30 days of follow-up.

**Supplementary Table 9.** The risk of heart failure according to the polygenic risk score in patients with atrial fibrillation, excluding the incident heart failure that occurred within the first 90 days of follow-up.

**Supplementary Table 1.** Definitions of covariates and outcomes.

| **Diagnosis** | **UK Biobank Field ID** | **Diagnostic code (ICD-9 or ICD-10)** |
| --- | --- | --- |
| **Inclusion** |  |  |
| Atrial fibrillation | 41270, 41280, 131350 | I48 |
| **Primary outcome** |  |  |
| Heart failure | 41270, 41280, 131354 | I50, I11.0, I13.0, I13.2 |
| **Covariates** |  |  |
| Age | 21022 |  |
| Sex | 31 |  |
| Body mass index | 21001 |  |
| Current smoking | 20116 |  |
| Alcohol intake | 1558 |  |
| Diabetes mellitus | 2443, 6153, 6177, 20002, 130708, 130710, 130712, 130714 |  |
|  | 41270, 41280 | E11-E14 |
| Hypertension | 6153, 6177, 20002, 2966, 131286, 131288, 131290, 131292, 131294 |  |
|  | 41270, 41280 | I10-I13, I15 |
| Dyslipidemia | 6153, 6177, 20002, 130814 |  |
|  | 41270, 41280 | E78 |
| Myocardial infarction | 41270, 41280 | I21-I22, I25 |
| Stroke | 20002, 20008, 20004, 20010, 41270, 41280, 131360, 131362, 131364, 131366, 131368, 41271, 41281, 41272, 41282, 42006 | I60-I64 |
| Any cardiomyopathy | 20002 | 1079, 1588 |
|  | 41270, 41280 | I42 |
| Townsend Deprivation Index | 189 |  |

Abbreviations: ICD, International Classification of Diseases.

**Supplementary Table 2.** Baseline characteristics of patients with atrial fibrillation included in the study stratified by age and polygenic risk score for heart failure.

|  | Total | | p-value | Age < 60 years | | p-value | Age ≥ 60 years | | p-value |
| --- | --- | --- | --- | --- | --- | --- | --- | --- | --- |
|  | Low PRS  for HF | Mod-High PRS  for HF |  | Low PRS  for HF | Mod-High PRS  for HF |  | Low PRS  for HF | Mod-High PRS  for HF |  |
|  | N=7,056 (33.3%) | N=14,111 (66.7%) |  | N=700 (31.4%) | N=1,531 (68.6%) |  | N=6,356 (33.6%) | N=12,580 (66.4%) |  |
| Age (years) | 69.3 ± 6.8 | 68.9 ± 6.9 | <0.001 | 55.2 ± 3.8 | 55.1 ± 3.9 | 0.82 | 70.8 ± 5.1 | 70.6 ± 5.0 | 0.004 |
| Male | 4,274 (60.6%) | 8,633 (61.2%) | 0.39 | 445 (63.6%) | 1,033 (67.5%) | 0.071 | 3,829 (60.2%) | 7,600 (60.4%) | 0.82 |
| Body mass index (kg/m2) | 28.4 ± 5.1 | 29.0 ± 5.3 | <0.001 | 28.5 ± 5.5 | 29.7 ± 6.3 | <0.001 | 28.4 ± 5.1 | 28.9 ± 5.2 | <0.001 |
| Household income before tax (pound) |  |  | 0.67 |  |  | 0.39 |  |  | 0.28 |
| <18,000 | 1,814 (25.7%) | 3,763 (26.7%) |  | 108 (15.4%) | 235 (15.3%) |  | 1,706 (26.8%) | 3,528 (28.0%) |  |
| 18,000 - 30,999 | 1,723 (24.4%) | 3,384 (24.0%) |  | 103 (14.7%) | 273 (17.8%) |  | 1,620 (25.5%) | 3,111 (24.7%) |  |
| 31,000 - 51,999 | 1,265 (17.9%) | 2,466 (17.5%) |  | 186 (26.6%) | 399 (26.1%) |  | 1,079 (17.0%) | 2,067 (16.4%) |  |
| 52,000 - 100,000 | 775 (11.0%) | 1,504 (10.7%) |  | 169 (24.1%) | 375 (24.5%) |  | 606 (9.5%) | 1,129 (9.0%) |  |
| >100,000 | 194 (2.7%) | 386 (2.7%) |  | 52 (7.4%) | 98 (6.4%) |  | 142 (2.2%) | 288 (2.3%) |  |
| Comorbidities |  |  |  |  |  |  |  |  |  |
| Hypertension | 4,288 (60.8%) | 9,011 (63.9%) | <0.001 | 293 (41.9%) | 672 (43.9%) | 0.37 | 3,995 (62.9%) | 8,339 (66.3%) | <0.001 |
| Myocardial infarction | 737 (10.4%) | 1,586 (11.2%) | 0.081 | 42 (6.0%) | 121 (7.9%) | 0.11 | 695 (10.9%) | 1,465 (11.6%) | 0.15 |
| Diabetes mellitus | 936 (13.3%) | 2,168 (15.4%) | <0.001 | 62 (8.9%) | 199 (13.0%) | 0.005 | 874 (13.8%) | 1,969 (15.7%) | <0.001 |
| Stroke | 649 (9.2%) | 1,262 (8.9%) | 0.54 | 30 (4.3%) | 83 (5.4%) | 0.26 | 619 (9.7%) | 1,179 (9.4%) | 0.42 |
| Cardiomyopathy | 69 (1.0%) | 142 (1.0%) | 0.844 | 11 (1.6%) | 26 (1.7%) | 0.828 | 58 (0.9%) | 116 (0.9%) | 0.948 |
| Medications |  |  |  |  |  |  |  |  |  |
| Hypertension medications | 2,631 (37.3%) | 5,717 (40.5%) | <0.001 | 138 (19.7%) | 337 (22.0%) | 0.22 | 2,493 (39.2%) | 5,380 (42.8%) | <0.001 |
| Dyslipidemia medications | 2,074 (29.4%) | 4,583 (32.5%) | <0.001 | 94 (13.4%) | 262 (17.1%) | 0.027 | 1,980 (31.2%) | 4,321 (34.3%) | <0.001 |
| Lifestyle behaviors |  |  |  |  |  |  |  |  |  |
| Current smoking | 727 (10.3%) | 1,480 (10.5%) | 0.68 | 97 (13.9%) | 242 (15.8%) | 0.23 | 630 (9.9%) | 1,238 (9.8%) | 0.88 |
| Daily drinking | 1759 (25.5%) | 3332 (24.1%) | 0.031 | 144 (21.0%) | 303 (20.1%) | 0.612 | 1615 (26.0%) | 3029 (24.6%) | 0.042 |
| Townsend Deprivation Index | -1.4 ± 3.0 | -1.4 ± 3.1 | 0.28 | -1.0 ± 3.2 | -1.1 ± 3.2 | 0.81 | -1.5 ± 3.0 | -1.4 ± 3.0 | 0.25 |
| CHA₂DS₂-VASc | 2.4 ± 1.4 | 2.4 ± 1.4 | 0.65 | 1.0 ± 0.9 | 1.1 ± 1.0 | 0.15 | 2.6 ± 1.3 | 2.6 ± 1.3 | 0.37 |

**Supplementary Table 3.** The risk of heart failure according to the increment of one-standard deviation of heart failure polygenic risk score in patients with atrial fibrillation stratified by age.

| **Group** | **Adjusted HR (95% CI)** | | |
| --- | --- | --- | --- |
|  | **Model 1** | **Model 2** | **Model 3** |
| Total | | | |
| HF PRS (per 1 SD increase) | 1.101 (1.051-1.153) | 1.111 (1.060-1.164) | 1.096 (1.040-1.154) |
|  | p<0.001 | p<0.001 | p=0.001 |
| Age<60 | | | |
| HF PRS (per 1 SD increase) | 1.255 (1.051-1.499) | 1.253 (1.047-1.500) | 1.290 (1.064-1.563) |
|  | p=0.012 | p=0.014 | p=0.010 |
| Age≥60 | | | |
| HF PRS (per 1 SD increase) | 1.093 (1.042-1.147) | 1.101 (1.050-1.156) | 1.082 (1.025-1.142) |
|  | p<0.001 | p<0.001 | p=0.004 |

Abbreviations: HR, hazard ratio; CI, confidence interval; HF, heart failure; PRS, polygenic risk score; SD, standard deviation

Model 1 was adjusted by chip and genetic principal component 1-10.

Model 2 was adjusted by age, sex, enrollment center, chip, and genetic principal component 1-10.

Model 3 was adjusted by age, sex, enrollment center, body mass index, household income before tax, hypertension, myocardial infarction, diabetes mellitus, stroke, any cardiomyopathy, chip, and genetic principal component 1-10.

**Supplementary Table 4**. Reclassification of predicted three-year risk of heart failure by adding genetic risk group on clinical risk factor model.

| Predicted risk by clinical risk factor model ^a)^ | Predicted risk by new model  (clinical risk factor model + genetic risk group) | | | | | | | Proportion of reclassification as | | Net % correctly reclassified | Category-based NRI (95% CI) |
| --- | --- | --- | --- | --- | --- | --- | --- | --- | --- | --- | --- |
|  | <2.5% | 2.5 to <5.0% | 5.0 to <7.5% | 7.5 to <10.0% | 10.0 to <12.5% | 12.5 to <15.0% | ≥15.0% | Increased risk | Decreased risk |  |  |
| Age <60 |  |  |  |  |  |  |  |  |  |  |  |
| 3-year HF (+) |  |  |  |  |  |  |  | 35.5% | 14.0% | 21.5% | 20.4% (6.7%-34.1%) |
| <2.5% | 6 | 2 | 0 | 0 | 0 | 0 | 0 |  |  |  |  |
| 2.5 to <5.0% | 1 | 19 | 8 | 0 | 0 | 0 | 0 |  |  |  |  |
| 5.0 to <7.5% | 0 | 1 | 12 | 12 | 0 | 0 | 0 |  |  |  |  |
| 7.5 to <10.0% | 0 | 4 | 1 | 4 | 4 | 0 | 0 |  |  |  |  |
| 10.0 to <12.5% | 0 | 0 | 4 | 0 | 4 | 6 | 0 |  |  |  |  |
| 12.5 to <15.0% | 0 | 0 | 2 | 1 | 0 | 2 | 6 |  |  |  |  |
| ≥15.0% | 0 | 0 | 0 | 0 | 1 | 0 | 7 |  |  |  |  |
| 3-year HF (-) |  |  |  |  |  |  |  | 23.0% | 21.9% | -1.1% |  |
| <2.5% | 265 | 76 | 0 | 0 | 0 | 0 | 0 |  |  |  |  |
| 2.5 to <5.0% | 216 | 440 | 148 | 0 | 0 | 0 | 0 |  |  |  |  |
| 5.0 to <7.5% | 0 | 103 | 198 | 94 | 0 | 0 | 0 |  |  |  |  |
| 7.5 to <10.0% | 0 | 29 | 19 | 69 | 54 | 0 | 0 |  |  |  |  |
| 10.0 to <12.5% | 0 | 0 | 20 | 0 | 17 | 37 | 1 |  |  |  |  |
| 12.5 to <15.0% | 0 | 0 | 0 | 8 | 0 | 7 | 20 |  |  |  |  |
| ≥15.0% | 0 | 0 | 0 | 3 | 8 | 3 | 36 |  |  |  |  |
| Age ≥60 |  |  |  |  |  |  |  |  |  |  |  |
| 3-year HF (+) |  |  |  |  |  |  |  | 10.5% | 9.4% | 1.1% | 1.8% (0.0%-4.1%) |
| <2.5% | 0 | 0 | 0 | 0 | 0 | 0 | 0 |  |  |  |  |
| 2.5 to <5.0% | 0 | 30 | 8 | 0 | 0 | 0 | 0 |  |  |  |  |
| 5.0 to <7.5% | 0 | 15 | 353 | 60 | 0 | 0 | 0 |  |  |  |  |
| 7.5 to <10.0% | 0 | 0 | 45 | 371 | 40 | 0 | 0 |  |  |  |  |
| 10.0 to <12.5% | 0 | 0 | 0 | 36 | 230 | 41 | 0 |  |  |  |  |
| 12.5 to <15.0% | 0 | 0 | 0 | 0 | 42 | 124 | 25 |  |  |  |  |
| ≥15.0% | 0 | 0 | 0 | 0 | 0 | 19 | 226 |  |  |  |  |
| 3-year HF (-) |  |  |  |  |  |  |  | 9.1% | 9.9% | 0.8% |  |
| <2.5% | 0 | 0 | 0 | 0 | 0 | 0 | 0 |  |  |  |  |
| 2.5 to <5.0% | 0 | 611 | 170 | 0 | 0 | 0 | 0 |  |  |  |  |
| 5.0 to <7.5% | 0 | 327 | 5478 | 629 | 0 | 0 | 0 |  |  |  |  |
| 7.5 to <10.0% | 0 | 0 | 636 | 3999 | 355 | 0 | 0 |  |  |  |  |
| 10.0 to <12.5% | 0 | 0 | 0 | 355 | 1644 | 250 | 0 |  |  |  |  |
| 12.5 to <15.0% | 0 | 0 | 0 | 0 | 223 | 831 | 114 |  |  |  |  |
| ≥15.0% | 0 | 0 | 0 | 0 | 0 | 110 | 1008 |  |  |  |  |

Abbreviations: CI, confidence interval; HF, heart failure; NRI, net reclassification improvement.

1. Clinical risk factor models are Model B for age <60 and Model C for age ≥60, which are described in Table 3.

Model B: male, body mass index, household income before tax, myocardial infarction, hypertension medication, and dyslipidemia medication.

Model C: age, male, body mass index, hypertension, myocardial infarction, diabetes mellitus, any cardiomyopathy, current smoking, and Townsend Deprivation Index.

**Supplementary Table 5.** Subgroup analysis of the risk of heart failure according to the polygenic risk score in patients with atrial fibrillation.

|  |  | **Total** | | | | | | **Age<60** | | | | |
| --- | --- | --- | --- | --- | --- | --- | --- | --- | --- | --- | --- | --- |
| **Subgroup** | **PRS for HF** | **Event / N** | **Cumulative incidence**^a)^ | **Mean years from AF to HF** | **Adjusted HR**  **(95% CI)^a)^** | **P-value** | **P-for-interaction** | **Mean years from AF to HF** | **Adjusted HR**  **(95% CI)^a)^** | **P-value** | **P-for-interaction** | **P-for-interaction,  comparison with adjusted HR of mod-high PRS in age≥60** |
|  |  |  |  |  | **Model 3** |  |  |  | **Model 3** |  |  |  |
| **Sex** | |  |  |  |  |  |  |  |  |  |  |  |
| Female | Low PRS  for HF | 314/2782 | 8.2% | 1.74 (0.36-4.72) | 1 (Reference) | 0.271 | 0.428 | 5.01 (1.12-7.05) | 1 (Reference) | 0.572 | 0.231 | 0.717 |
|  | Mod-High PRS  for HF | 706/5478 | 9.3% | 1.90 (0.43-4.89) | 1.118 (0.917-1.363) |  |  | 2.39 (0.85-5.27) | 1.322 (0.503-3.476) |  |  |  |
| Male | Low PRS  for HF | 541/4274 | 9.3% | 1.97 (0.37-4.75) | 1 (Reference) | 0.010 |  | 3.75 (0.74-6.96)^b)^ | 1 (Reference) | 0.003 |  | 0.013 |
|  | Mod-High PRS  for HF | 1297/8633 | 11.3% | 1.63 (0.36-4.32) | 1.198 (1.044-1.375) |  |  | 1.24 (0.32-4.37)^b)^ | 2.584 (1.396-4.782) |  |  |  |
| **Hypertension** | |  |  |  |  |  |  |  |  |  |  |  |
| No | Low PRS  for HF | 243/2768 | 6.5% | 1.42 (0.30-4.72) | 1 (Reference) | 0.042 | 0.499 | 3.92 (0.77-6.27)^b)^ | 1 (Reference) | 0.006 | 0.244 | 0.016 |
|  | Mod-High PRS  for HF | 539/5100 | 7.7% | 1.50 (0.32-4.61) | 1.242 (1.008-1.530) |  |  | 0.93 (0.25-3.68)^b)^ | 3.091 (1.388-6.881) |  |  |  |
| Yes | Low PRS  for HF | 612/4288 | 10.4% | 2.12 (0.37-4.75) | 1 (Reference) | 0.040 |  | 4.45 (0.96-7.40)^b)^ | 1 (Reference) | 0.138 |  | 0.319 |
|  | Mod-High PRS  for HF | 1464/9011 | 12.1% | 1.81 (0.38-4.58) | 1.151 (1.006-1.316) |  |  | 2.46 (0.45-5.45)^b)^ | 1.666 (0.849-3.269) |  |  |  |
| **Diabetes mellitus** | |  |  |  |  |  |  |  |  |  |  |  |
| No | Low PRS  for HF | 678/6120 | 7.8% | 2.01 (0.39-5.00) | 1 (Reference) | 0.003 | 0.305 | 4.27 (0.81-6.86)^b)^ | 1 (Reference) | 0.005 | 0.637 | 0.021 |
|  | Mod-High PRS  for HF | 1549/11943 | 9.5% | 1.69 (0.36-4.66) | 1.216 (1.070-1.382) |  |  | 1.32 (0.32-4.57)^b)^ | 2.234 (1.277-3.908) |  |  |  |
| Yes | Low PRS  for HF | 177/936 | 16.1% | 1.45 (0.24-3.71) | 1 (Reference) | 0.578 |  | 2.88 (1.08-7.57) | 1 (Reference) | 0.191 |  | 0.412 |
|  | Mod-High PRS  for HF | 454/2168 | 16.4% | 1.78 (0.38-4.26) | 1.071 (0.842-1.362) |  |  | 3.14 (0.45-5.45) | 3.144 (0.565-17.500) |  |  |  |
| **Myocardial infarction** | |  |  |  |  |  |  |  |  |  |  |  |
| No | Low PRS  for HF | 692/6319 | 7.9% | 1.79 (0.36-4.82) | 1 (Reference) | 0.001 | 0.042 | 4.27 (0.81-7.14)^b)^ | 1 (Reference) | 0.009 | 0.745 | 0.052 |
|  | Mod-High PRS  for HF | 1667/12525 | 9.7% | 1.75 (0.38-4.65) | 1.246 (1.099-1.414) |  |  | 1.55 (0.33-4.67)^b)^ | 2.055 (1.193-3.539) |  |  |  |
| Yes | Low PRS  for HF | 163/737 | 17.1% | 1.97 (0.35-4.16) | 1 (Reference) | 0.554 |  | 2.88 (1.65-5.02) | 1 (Reference) | 0.085 |  | 0.126 |
|  | Mod-High PRS  for HF | 336/1586 | 16.8% | 1.51 (0.30-4.19) | 0.925 (0.715-1.197) |  |  | 1.52 (0.32-5.75) | 4.423 (0.813-24.064) |  |  |  |

Abbreviations: PRS, polygenic risk score; HF, heart failure; AF, atrial fibrillation; HR, hazard ratio; CI, confidence interval; Mod, moderate.

a) 3-year cumulative incidence and hazard ratios

b) p<0.05

Model 3 was adjusted by age, sex, enrollment center, body mass index, household income before tax, hypertension, myocardial infarction, diabetes mellitus, stroke, any cardiomyopathy, chip, and genetic principal component 1-10.

**Supplementary Table 6.** The risk of heart failure according to the polygenic risk score in patients with atrial fibrillation, excluding cardiomyopathies stratified by age.

| **Group** | **Event / N^a)^** | **Cumulative incidence^b)^** | **Mean years from AF to HF** | **Adjusted HR (95% CI)^b)^** | | |
| --- | --- | --- | --- | --- | --- | --- |
|  |  |  |  | **Model 1** | **Model 2** | **Model 3** |
| **Total** | | | | | | |
| Low PRS for HF | 780/6901 | 8.3% | 2.90 (2.69-3.12) | 1 (Reference) | 1 (Reference) | 1 (Reference) |
| Mod-High PRS for HF | 1823/13751 | 9.8% | 2.77 (2.64-2.90) | 1.192 (1.071-1.326) | 1.207 (1.084-1.343) | 1.143 (1.016-1.287) |
|  |  |  | p=0.275 | p=0.001 | p=0.001 | p=0.026 |
| **Age<60** |  |  |  |  |  |  |
| Low PRS for HF | 40/672 | 2.2% | 4.79 (3.66-5.93) | 1 (Reference) | 1 (Reference) | 1 (Reference) |
| Mod-High PRS for HF | 124/1471 | 5.6% | 2.74 (2.22-3.25) | 2.660 (1.503-4.708) | 2.625 (1.483-4.647) | 2.421 (1.338-4.380) |
|  |  |  | p<0.001 | p=0.001 | p=0.001 | p=0.004 |
| **Age≥60** | | | | | | |
| Low PRS for HF | 740/6229 | 9.0% | 2.80 (2.59-3.02) | 1 (Reference) | 1 (Reference) | 1 (Reference) |
| Mod-High PRS for HF | 1699/12280 | 10.3% | 2.77 (2.64-2.91) | 1.153 (1.034-1.286) | 1.163 (1.043-1.297) | 1.097 (0.972-1.239) |
|  |  |  | 0.812 | p=0.010 | p=0.007 | p=0.133 |
|  |  |  |  | p-for-interaction=0.007 | p-for-interaction=0.007 | p-for-interaction=0.019 |

Abbreviations: AF, atrial fibrillation; HF, heart failure; HR, hazard ratio; CI, confidence interval; PRS, polygenic risk score.

^a)^ Observed events during total follow-up period

^b)^ 3-year cumulative incidence and hazard ratios.

Model 1 was adjusted by chip and genetic principal component 1-10.

Model 2 was adjusted by age, sex, enrollment center, chip, and genetic principal component 1-10.

Model 3 was adjusted by age, sex, enrollment center, body mass index, household income before tax, hypertension, myocardial infarction, diabetes mellitus, stroke, chip, and genetic principal component 1-10.

**Supplementary Table 7.** Harrell's C-statistics and net reclassification improvement of polygenic risk score in predicting heart failure in patients with atrial fibrillation, excluding cardiomyopathies.

|  | **Harrell's C-statistics (95% CI)** | **p-value** | **NRI (%)** | **p-value** |
| --- | --- | --- | --- | --- |
| Total |  |  |  |  |
| Model A | 0.646 (0.631-0.660), p<0.001 | - | ref. | - |
| Model A + PRS for HF (low vs. mod-high) | 0.647 (0.632-0.662), p<0.001 | 0.161 | 7.69 (2.06-13.32) | 0.007 |
| Age <60 |  |  |  |  |
| Model B | 0.704 (0.650-0.759), p<0.001 | - | ref. | - |
| Model B + PRS for HF (low vs. mod-high) | 0.734 (0.687-0.781), p<0.001 | 0.012 | 33.44 (11.94-54.94) | 0.002 |
| Age ≥60 |  |  |  |  |
| Model C | 0.633 (0.618-0.647), p<0.001 | - | ref. | - |
| Model C + PRS for HF (low vs. mod-high) | 0.634 (0.620-0.649), p<0.001 | 0.091 | 7.06 (1.81-12.31) | 0.009 |

Abbreviations: NRI, net reclassification improvement; CI, confidence interval; PRS, polygenic risk score; HF, heart failure; mod, moderate

Model A: age, male, body mass index, household income before tax, hypertension medication, myocardial infarction, diabetes mellitus, Townsend Deprivation Index, and current smoking.

Model B: male, body mass index, household income before tax, myocardial infarction, hypertension medication, and dyslipidemia medication.

Model C: age, male, body mass index, hypertension, myocardial infarction, diabetes mellitus, current smoking, and Townsend Deprivation Index.

Clinical features of Model A, B, and C to predict heart failure in patients with atrial fibrillation were selected from stepwise Cox regression analysis in each age group (total vs. age <60 years vs. age ≥60 years) among the following covariate: age, sex, body mass index (BMI), household income before tax, hypertension (HTN), myocardial infarction (MI), diabetes mellitus (DM), stroke, current smoking, daily drinking, HTN medication, dyslipidemia medication, and Townsend Deprivation Index (TDI).

**Supplementary Table 8.** The risk of heart failure according to the polygenic risk score in patients with atrial fibrillation, excluding the incident heart failure that occurred within the first 30 days of follow-up.

| **Group** | **Event / N ^a)^** | **Cumulative incidence ^b)^** | **Adjusted HR (95% CI) ^b)^** | | |
| --- | --- | --- | --- | --- | --- |
|  |  |  | **Model 1** | **Model 2** | **Model 3** |
| Low PRS for HF | 745/7056 | 7.5% | 1 (Reference) | 1 (Reference) | 1 (Reference) |
| Mod-High PRS for HF | 1773/14111 | 9.1% | 1.241 (1.107-1.391) | 1.255 (1.120-1.406) | 1.211 (1.068-1.374) |
|  |  |  | p<0.001 | p<0.001 | p=0.003 |
| **Age<60** |  |  |  |  |  |
| Low PRS for HF | 55/700 | 3.4% | 1 (Reference) | 1 (Reference) | 1 (Reference) |
| Mod-High PRS for HF | 131/531 | 5.2% | 1.636 (1.014-2.640) | 1.613 (1.000-2.603) | 1.878 (1.085-3.250) |
|  |  |  | p=0.044 | p=0.050 | p=0.024 |
| **Age≥60** | | | | | |
| Low PRS for HF | 690/6356 | 8.0% | 1 (Reference) | 1 (Reference) | 1 (Reference) |
| Mod-High PRS for HF | 1642/12580 | 9.7% | 1.225 (1.089-1.377) | 1.235 (1.098-1.389) | 1.176 (1.033-1.339) |
|  |  |  | p=0.001 | p<0.001 | p=0.014 |
|  |  |  | p-for-interaction=0.335 | p-for-interaction=0.360 | p-for-interaction=0.097 |

Abbreviations: AF, atrial fibrillation; HF, heart failure; HR, hazard ratio; CI, confidence interval; PRS, polygenic risk score.

^a)^ Observed events during the total follow-up period

^b)^ 3-year cumulative incidence and hazard ratios.

Model 1 was adjusted by chip and genetic principal component 1-10.

Model 2 was adjusted by age, sex, enrollment center, chip, and genetic principal component 1-10.

Model 3 was adjusted by age, sex, enrollment center, body mass index, household income before tax, hypertension, myocardial infarction, diabetes mellitus, stroke, any cardiomyopathy, chip, and genetic principal component 1-10.

**Supplementary Table 9.** The risk of heart failure according to the polygenic risk score in patients with atrial fibrillation, excluding the incident heart failure that occurred within the first 90 days of follow-up.

| **Group** | **Event / N ^a)^** | **Cumulative incidence ^b)^** | **Adjusted HR (95% CI) ^b)^** | | |
| --- | --- | --- | --- | --- | --- |
|  |  |  | **Model 1** | **Model 2** | **Model 3** |
| Low PRS for HF | 676/7056 | 6.8% | 1 (Reference) | 1 (Reference) | 1 (Reference) |
| Mod-High PRS for HF | 1596/14111 | 8.2% | 1.223 (1.083-1.382) | 1.236 (1.094-1.396) | 1.211 (1.058-1.385) |
|  |  |  | p=0.001 | p=0.001 | p=0.005 |
| **Age<60** |  |  |  |  |  |
| Low PRS for HF | 54/700 | 3.3% | 1 (Reference) | 1 (Reference) | 1 (Reference) |
| Mod-High PRS for HF | 121/531 | 4.8% | 1.548 (0.946-2.534) | 1.528 (0.933-2.502) | 1.763 (0.997-3.118) |
|  |  |  | p=0.082 | p=0.092 | p=0.051 |
| **Age≥60** | | | | | |
| Low PRS for HF | 622/6356 | 7.3% | 1 (Reference) | 1 (Reference) | 1 (Reference) |
| Mod-High PRS for HF | 1475/12580 | 8.7% | 1.208 (1.065-1.370) | 1.218 (1.074-1.381) | 1.179 (1.026-1.355) |
|  |  |  | p=0.003 | P=0.002 | p=0.020 |
|  |  |  | p-for-interaction=0.424 | p-for-interaction=0.451 | p-for-interaction=0.167 |

Abbreviations: AF, atrial fibrillation; HF, heart failure; HR, hazard ratio; CI, confidence interval; PRS, polygenic risk score.

^a)^ Observed events during the total follow-up period

^b)^ 3-year cumulative incidence and hazard ratios.

Model 1 was adjusted by chip and genetic principal component 1-10.

Model 2 was adjusted by age, sex, enrollment center, chip, and genetic principal component 1-10.

Model 3 was adjusted by age, sex, enrollement center, body mass index, household income before tax, hypertension, myocardial infarction, diabetes mellitus, stroke, any cardiomyopathy, chip, and genetic principal component 1-10.
